# Experiences, expectations and preferences regarding MRI and mammography as breast cancer screening tools in women at familial risk

**DOI:** 10.1016/j.breast.2021.01.002

**Published:** 2021-01-18

**Authors:** H. Amarens Geuzinge, Eveline A.M. Heijnsdijk, Inge-Marie Obdeijn, Harry J. de Koning, Madeleine M.A. Tilanus-Linthorst, on behalf of the FaMRIsc study group

**Affiliations:** a Department of Public Health, Erasmus MC, University Medical Centre Rotterdam, Rotterdam, the Netherlands; b Department of Radiology and Nuclear Medicine, Erasmus MC, Erasmus University Medical Centre, Rotterdam, the Netherlands; c Department of Surgery, Erasmus MC, University Medical Centre Rotterdam, Rotterdam, the Netherlands

**Keywords:** Breast neoplasms, MRI, Mammography, Early detection of cancer, Patient satisfaction, Patient preference

## Abstract

**Background:**

Several studies have investigated MRI breast cancer screening in women at increased risk, but little is known about their preferences. In this study, experiences, expectations and preferences for MRI and mammography were evaluated among women undergoing screening with MRI and/or mammography in the randomized FaMRIsc trial.

**Methods:**

A 17-item questionnaire was sent to 412 women in the FaMRIsc trial. Participants were aged 30–55 years, had a ≥20% cumulative lifetime risk, but no *BRCA1/2* or *TP53* gene variant, and were screened outside the population-based screening program. Women received annual mammography (mammography-group), or annual MRI and biennial mammography (MRI-group). We asked whether women trust the screening outcome, what they consider as (dis)advantages, which screening they prefer and what they expect of the early detection by the screening tools.

**Results:**

255 (62%) women completed our questionnaire. The high chance of early cancer detection was the most important advantage of MRI screening (MRI-group: 95%; mammography-group: 74%), while this was also the main advantage of mammography (MRI-group: 57%; mammography-group: 72%). Most important disadvantages of MRI were the small tunnel and the contrast fluid (for 23–36%), and of mammography were its painfulness and X-radiation (for 48–60%). Almost the whole MRI-group and half the mammography-group preferred screening with MRI (either alone or with mammography).

**Discussion:**

Most women would prefer screening with MRI. The way women think of MRI and mammography is influenced by the screening strategy they are undergoing. Our outcomes can be used for creating information brochures when MRI will be implemented for more women.

## Highlights

• Women consider the small tunnel and contrast fluid important disadvantages of MRI.
• How women think of MRI is influenced by the screening strategy they are undergoing.
• Most women with a family history of breast cancer prefer screening with MRI.

## 1 Introduction

Breast cancer is the most common cancer among women. Many countries offer breast cancer screening in order to detect breast cancer at an early stage. High risk women are often offered breast cancer screening with mammography and/or MRI outside population-based screening programs [[1], [2], [3]]. Recently, research on MRI screening efficacy is extended to other subgroups of women with increased risk. A large randomized controlled trial in women with extremely dense breast tissue in the population-based screening program showed that additional MRI led to less interval cancers [4]. Another randomized controlled trial, investigating MRI screening in women with a family history of breast cancer but without a pathogenic gene variant (the FaMRIsc trial), also showed a higher sensitivity of MRI, and cancers being detected at an earlier stage, compared to mammography [5]. Unfortunately, MRI screening also leads to more false positive screening results, which was also seen in these trials [4,5], and it is more expensive than mammography [8,9].

Whether women should be screened with MRI is mostly based on cohort studies, randomized controlled trials and cost-effectiveness analyses. Little is known about preferences of women themselves. However, participant acceptability is crucial for a possible implementation of MRI screening. Due to the above-mentioned randomized trials, and increasing MRI expertise and technologic advances over the years, it is possible that this modality will be implemented for a greater amount of women in the future [10].

Several studies on population-based mammography screening showed that women regard the possibility of an earlier diagnosis as more important than the risk of false-positive screening results or overdiagnosis [11]. A study by Phillips et al. investigated patient preferences and attitudes towards contrast-enhanced spectral mammography (CESM) and MRI, and found that most high-risk women in their study preferred CESM over MRI if the exams had equal sensitivities [12]. Another study also showed a preference towards CESM over MRI [13]. In contrast, a study by Essink-Bot et al. showed that women with an increased risk for breast cancer undergoing MRI screening mainly preferred MRI as a screening test over mammography when assuming equal performance [14]. They also showed that these women experienced ‘lying in the tunnel’, ‘noise of the machine’ and the fact that they were not allowed to move during the procedure as important burdens of MRI [14]. To our knowledge, no previous studies investigated what women who were randomized to either MRI or mammography screening expect and think of both tools.

In our study, we compare experiences, expectations and preferences for MRI and mammography among women with a family history of breast cancer who were either screened with mammography or with a combination of MRI and mammography during the FaMRIsc trial [5,15].

## 2 Methods

### 2.1 Study population

The Familial MRI Screening (FaMRIsc) trial was a multicenter randomized controlled trial assessing the efficacy of MRI screening in comparison to mammography in women with a family history of breast cancer, and assessing the influence of breast density [15]. Women aged 30–55 years with a cumulative lifetime risk for breast cancer of ≥20% due to a family history without a known *BRCA1/2* or *TP53* variant were randomly assigned to two groups: 1) the mammography-group: screening consisting of annual mammography, and 2) the MRI-group: screening consisting of annual MRI and biennial mammography. Both groups also received annual clinical breast examination (CBE). Women who did not want to be randomized but who provided consent for registration of their screening results were grouped as the registration group (231/1586 = 15%) and could either be screened according to the mammography-protocol (218 of 231 = 94%) or MRI-protocol (13 of 231 = 6%) [15]. In this paper, all women who were screened according to the MRI-protocol are referred to as the MRI-group, and women screened according to the mammography-protocol are referred to as the mammography-group.

During the final months of the FaMRIsc trial (end of 2017), 412 of 1586 (26%) participants were sent a letter in which they were asked to complete a questionnaire [15]. The letter contained a code to log in to a website to complete the questionnaire. Participants could also request a printed version of the questionnaire. The invitation letter for the questionnaire was sent randomly to an equal number of women per screening protocol who filled in a previous questionnaire. This was done to increase the likelihood of reaching a high response rate. We aimed to also include all women with a breast cancer diagnosis, so we also invited participants who were diagnosed with an invasive cancer during the trial. In the letter we highlighted that we were not testing their knowledge but that we were interested in their opinion.

### 2.2 Questionnaire

The questionnaire was developed by three researchers of the FaMRIsc study (of which one was also a clinician) and was discussed in a group of five other breast cancer screening researchers. The questionnaire was sent to women in November 2017. In January 2018, a reminder was sent to all women who did not respond to the first invitation.

The questionnaire contained 17 questions and an open space to fill in the year of birth. The questions included in this paper encompassed four categories: 1) breast cancer (screening) history; 2) advantages and disadvantages; 3) expectations; 4) preference. The questionnaire can be found in the Supplementary Appendix. Questions 1–4 were related to the history of breast cancer screening and a possible breast cancer diagnosis. The category ‘advantages and disadvantages’ consisted of four questions (questions 11, 12, 13 and 14), all containing a list of advantages or disadvantages of MRI or mammography screening. In these questions, participants were asked to assign all options from the list that they consider important advantages or disadvantages of MRI and mammography. In case multiple answers were chosen, participants were asked to indicate which answer was most important to them. The category ‘expectations’ contained a question about screening in general (question 5), a question about early detection of MRI in comparison with mammography (question 10), and two questions about trust in the findings of mammography and MRI (questions 8 and 9), both on a Likert scale with a range of 0–4. The category ‘preference’ contained a question to obtain participants’ preferences for a screening modality and questions evaluating preference with regard to the ability of early detection, the chance of false-positive results and costs (question 6, 15, 16 and 17). One question was neither about mammography or MRI (question 7), and was therefore not included in this manuscript (but the outcomes can be found in the Supplementary appendix, table S9).

### 2.3 Statistical analyses

Outcomes were stratified according to the screening protocols of the study: the MRI-group and the mammography-group. Furthermore, outcomes of the preference for a screening modality (question 6) were stratified by women experiencing a false alarm yes/no (question 3), and by women who had ever had undergone MRI yes/no (question 4) when participating in the mammography-group. The latter stratification was also performed for the question about trust in the findings of MRI (question 9). Fisher’s exact tests were performed to examine differences in the answers between the groups. A *p*-value of 0.05 or less was considered statistically significant. Missing data were taken into account when analyzing the data, and were included in the tables, however these were not included in the Fisher’s exact tests.

## 3 Results

A total of 412 participants of the FaMRIsc study were sent a letter in which we asked them to fill in our questionnaire. After receiving the first letter, 178 women filled in the questionnaire, and another 77 women filled it in after the reminder was sent. This resulted in a response rate of 62% (255/412). Two women did not answer the question regarding the screening protocol they were assigned to. Therefore, we excluded the outcomes of these two women from the analyses. Of the respondents, 43% (108/253) was screened according to the MRI-protocol, and 57% (145/253) according to the mammography-protocol. Most of these women (241/253: 95% underwent randomization to these protocols, and the other 12 women participated in the registration group. Of the women who were screened according to the mammography-protocol, 36% (49/145) previously had a breast MRI, either for diagnostics or for screening. Women in the MRI-group were on average 50 years old (SD:6.3), and women in the mammography-group 51 years old (SD:6.4). Seven respondents were diagnosed with breast cancer (MRI-group: 5; mammography-group: 2), of which six were screen-detected within the FaMRIsc study, and one was an interval cancer. Four women were diagnosed with a precursor of breast cancer (ductal carcinoma in situ) before the FaMRIsc study, and two women were undergoing additional diagnostic testing due to a positive screening result (both in the MRI-group). All outcomes of the questions regarding breast cancer (screening history) can be found in the supplementary appendix (tables S1-S4).

### 3.1 Advantages and disadvantages

Table 1 shows how often specific advantages and disadvantages of mammography and MRI were chosen per group. In the MRI-group more women called ‘the high chance of early detection’ an advantage or MRI than of mammography (95% vs. 57% respectively), while in the mammography-group the advantage of ‘high chance of early detection’ was given as frequently for MRI as for mammography (74% vs 72% respectively). In both groups, the high chance of early detection was the most frequently mentioned advantage for both mammography and MRI. The two groups also agreed on the most important disadvantages of mammography screening: 1) ‘it is painful’, 2) ‘radiation risk’, and 3) ‘it does not detect all breast cancers’. Women who chose the option ‘Other, …’ for the questions about advantages and disadvantages of MRI, mostly wrote that they never had a breast MRI and therefore did not know what to answer. The disadvantage ‘it causes a false alarm sometimes’ was not frequently chosen, neither for MRI nor for mammography (percentages ranging from 5% to 20%).

**Table 1:** Advantages and disadvantages of mammography and MRI.

**Advantages of mammography**	MRI-group (N = 108)	Mammography-group (N = 145)	**Disadvantages of mammography**	MRI-group (N = 108)	Mammography-group (N = 145)
High chance of early detection of breast cancer	61 (57%)	105 (72%)	It is painful	65 (60%)	83 (57%)
You can get the screening result quickly	58 (54%)	98 (68%)	You get X-radiation	52 (48%)	71 (49%)
It does not take much time	43 (40%)	67 (46%)	It does not detect all breast cancers	52 (48%)	46 (32%)
I was already familiar with mammography	30 (28%)	46 (32%)	I do not see disadvantages of mammography	16 (15%)	29 (20%)
It has a small chance of a false alarm	14 (13%)	20 (14%)	It causes a false alarm sometimes	22 (20%)	26 (18%)
I can get it close to where I live	9 (8%)	17 (12%)	Other, …	4 (4%)	6 (4%)
I do not see advantages of mammography	10 (9%)	10 (7%)	It takes long before I get the result	1 (1%)	5 (3%)
It is not expensive	8 (7%)	8 (6%)	It takes (too) much time	0	1 (1%)
Other, …	9 (8%)	7 (5%)	I have to take off my clothes	0	0

**Advantages of MRI**	MRI-group (N = 108)	Mammography-group (N = 145)	**Disadvantages of MRI**	MRI-group (N = 108)	Mammography-group (N = 145)
High chance of early detection of breast cancer	103 (95%)	107 (74%)	You have to lie in a small tunnel	39 (36%)	47 (32%)
You don’t get X-radiation	43 (40%)	60 (41%)	The infusion of contrast fluid is unpleasant	35 (32%)	34 (23%)
It does not cause pain	45 (42%)	55 (38%)	The noise is unpleasant	35 (32%)	31 (21%)
You can get the screening result quickly	19 (18%)	33 (23%)	It takes a lot of time	27 (25%)	22 (15%)
It has a small chance of a false alarm	27 (25%)	21 (15%)	I do not see disadvantages of MRI	26 (24%)	19 (13%)
Other, …	1 (1%)	20 (14%)	Other, …	10 (9%)	41 (28%)
I can keep some clothes on	7 (7%)	6 (4%)	I have to wait more than one day for the result	20 (19%)	14 (10%)
			Some contrast fluid may remain in my body, even though no side effects of this are known	16 (15%)	12 (8%)
			It is expensive	10 (9%)	18 (12%)
			It does not detect all breast cancers	12 (11%)	12 (8%)
			It causes a false alarm sometimes	11 (10%)	7 (5%)
			It is far from home which causes travel time	3 (3%)	2 (1%)

When asking the participants to indicate which advantage and disadvantage of mammography screening was most important for them, most women ranked the chance of early detection by mammography as the most important advantage (mammography-group: 65% (67/103); MRI-group: 53% (39/74)), and the fact that mammography can be painful as the most important disadvantage (mammography-group: 41% (46/111); MRI-group: 29% (23/80)). Women of the MRI-group ranked the disadvantages ‘radiation risk’ and ‘it does not detect all breast cancers’ also as important disadvantages of mammography, with 26% (21/80) and 28% (22/80) respectively. When it comes to advantages of MRI, both groups ranked the early detection of breast cancers as most important (mammography-group: 60% (61/101); MRI-group: 81% (69/85)). The groups also agreed on the most important disadvantage of MRI: ‘you have to lie in a small tunnel’ (mammography-group: 26% (24/91); MRI-group: 24% (20/85)).

Percentages are calculated with the number of women as denominator. Since women were allowed to choose more than one option, the sum of all percentages is higher than 100%.

### 3.2 Expectations

Less than 2% of the women did not expect the chance to detect breast cancer at an early stage to be higher with screening than without screening (see appendix). Similar proportions of both groups thought that MRI has a higher chance of detecting breast cancer in an early stage than mammography (MRI-group: 84%; mammography-group: 81%). However, more women of the MRI-group thought that MRI has a much higher chance of detecting breast cancer in an early stage than women of the mammography-group (Table 2). The difference in expectation was statistically non-significant (*p* = 0.098).

**Table 2:** The chance of detecting breast cancer early by MRI is […] than with mammography.

	MRI-group (N = 108)	Mammography-group (N = 145)
Much smaller	0	1 (1%)
Smaller	2 (2%)	2 (1%)
Similar	15 (14%)	22 (15%)
Slightly higher	39 (36%)	69 (48%)
Much higher	52 (48%)	48 (33%)
Missing	0	3 (2%)

In total, 85% of the MRI-group and 92% of the mammography-group had quite some trust or a lot of trust in mammography screening (Table 3). The proportion of women with a lot of trust in mammography was relatively large in the mammography-group, compared to the MRI-group (57% versus 37%). The difference in trust in mammography was statistically significant (*p* = 0.014) between the groups. Higher proportions of women had a lot of trust in MRI, compared to mammography. However, a similar proportion of the mammography-group had a lot of trust in MRI (61%) as they had in mammography (57%). A relatively high proportion of the MRI-group had a lot of trust in MRI compared to the mammography-group (82% versus 61%). The difference in trust in MRI was significantly different (*p* < 0.001) between the groups. Subgroup analyses of trust in the findings of MRI, stratified by prior experience with MRI are shown in Table S5. A higher proportion of women who had prior experience with breast MRI had a lot of trust in MRI (38/49: 78%) than women who never had a breast MRI (45/89: 51%).

**Table 3:** Trust in the findings of mammography and MRI

	Do you trust the finding that you do/do not have breast cancer after only mammography?		Do you trust the findings that you do/do not have breast cancer after only MRI?	
	MRI-group (N = 108)	Mammography-group (N = 145)	MRI-group (N = 108)	Mammography-group (N = 145)
No trust	4 (4%)	2 (1%)	0	0
A little trust	8 (7%)	4 (3%)	2 (2%)	2 (1%)
Neutral	4 (4%)	6 (4%)	1 (1%)	31 (21%)
Quite some trust	52 (48%)	50 (35%)	17 (16%)	13 (9%)
A lot of trust	40 (37%)	82 (57%)	88 (82%)	88 (61%)
Missing	0	1 (1%)	0	11 (8%)

### 3.3 Preference

Preference for a screening strategy varied significantly per screening group (*p* < 0.001), as shown in Table 4. Relatively few women of the MRI-group (6%) preferred screening with only mammography, and 31% of the mammography-group preferred this strategy. Half of the MRI-group (54 of 108) and approximately a third of the mammography-group (50 of 145) preferred screening with both MRI and mammography.

**Table 4:** Preference of screening modality.

	MRI-group (N = 108)	Mammography-group (N = 145)
Mammography	6 (6%)	45 (31%)
MRI	41 (38%)	26 (18%)
Mammography and MRI	54 (50%)	50 (35%)
In the national breast cancer screening program	0	5 (3%)
No preference	6 (6%)	8 (6%)
No screening at all	0	0
Other, …	1 (1%)	9 (6%)
Missing	0	2 (1%)

Subgroup analyses of preference by women who ever had a false alarm and those who had not, are shown in Table S6. Women within the mammography-group who ever had a false alarm, had slightly less often a preference for mammography only (27%), compared to women who never had a false alarm within the mammography-group (33%). Women within the MRI-group who ever had a false alarm had more often a preference for screening with MRI only (45%) compared to women who never had a false alarm (34%) in the MRI-group. It is important to mention that in these analyses we do not know by which screening tool (mammography or MRI) false alarms were caused in the MRI-group because the questionnaire was anonymous.

Table S7 shows the preference outcomes stratified by prior experience with MRI of women in the mammography-group. Of the women having prior experience with MRI, 18% had a preference for MRI screening and 39% preferred a combination of mammography and MRI. Of the women not having prior experience with MRI, also 18% had a preference for MRI, and 30% preferred a combination of mammography and MRI.

Answers to questions 15 and 16 showed that most women (i.e. 36-39%) in the MRI-group preferred screening with MRI regardless of how much better the early detection or the chance of getting a false-positive result are (see Tables S10, S11). 41% of the Mx-group preferred MRI screening in case MRI would case a false alarm as often as mammography. Only a few women (11–15%) preferred MRI screening if it causes a false alarm two or three times as often as mammography. Question 17 showed that for most women (approximately 75%), their preference for MRI or mammography seemed not influenced by its costs.

## 4 Discussion

Women who were screened with both MRI and mammography had a different view on these screening tools than women who were screened with mammography only. A higher proportion of women in the MRI-group valued the advantage of the high chance of early detection of MRI important compared to women in the mammography-group. Also, more women of the MRI-group thought that MRI has a much higher chance of detecting breast cancer in an early stage than women of the mammography-group. Furthermore, women screened with MRI plus mammography were having less trust in the results of a mammogram and more trust in the results of MRI than women screened with mammography only. The preference for screening strategy differed also between the two groups: almost all women of the MRI-group preferred screening with either MRI only or a combination of MRI and mammography, whereas half of the mammography-group preferred a screening strategy with MRI. Most participants in our study understood the aim of screening very well and indicated the early diagnosis of breast cancer by mammography and MRI as the most important advantage. This is in line with previous studies in women at average breast cancer risk when choosing between mammography and no screening, showing that early diagnosis is of most importance to them [11]. A previous study, during the early days of MRI screening, with a sample of 178 high risk women all undergoing mammography and MRI, showed that 44% preferred MRI as a screening test and 14% preferred mammography when equal performance of these tests was assumed. Furthermore, they showed that 64% of the participants was completely reassured by a negative MRI test result, but only 40% for mammography [14]. In our study, trust in MRI was higher: 82% and 61% of the MRI-group and mammography-group respectively had a lot of trust in the findings of MRI. Fewer women of the MRI-group (37%) had a lot of trust in mammography compared to the mammography-group (57%).

This study has some strengths and limitations. A strength of this study is the fact that two groups of women filled in our questionnaire, so answers by women who were screened with MRI and mammography could be compared with answers by women who were not screened with MRI. To our knowledge, previous studies focused on women who were all screened with both screening modalities. A limitation of this study is the fact that the questionnaire was not pilot tested. Especially in the Mammography-group, women indicated that they were not able to say what they thought were advantages and disadvantages of MRI, since they never had an MRI. By pilot testing the questionnaire we may have been able to prevent this by stating these questions differently. Another limitation of our study is the risk of response bias. Women with a strong opinion on MRI and mammography may have filled in the questionnaire more often than women who did not have a strong opinion. As the response rates were different between the two groups, a response bias may have been the case in our study. A third limitation is the fact that overdiagnosis was not listed in our questionnaire. However, in the literature we found that women have limited awareness of overdiagnosis [11].

When interpreting the outcomes on respondents perception on the price of MRI, it is important to mention that costs of the MRI were provided by the FaMRIsc study. In case women were referred for further assessment after a positive MRI, insurers were billed for these costs. In case MRI becomes part of screening guidelines in the Netherlands, the costs of MRI will also be billed to insurers. Currently, in the Netherlands, people pay a deductible of at least 385 euros per year, so therefore sometimes women will have to pay for the MRI themselves when MRI is implemented.

Almost all respondents (95%) were randomly assigned to one of the two screening protocols. In case women did not want to be screened according to the MRI protocol, we assume they would have refused randomization. However, we do not know whether respondents in the Mammography-group would have accepted MRI screening eventually, and we did not ask this in our questionnaire. Therefore, we cannot tell which of the disadvantages of MRI would be reasons for non-participation. A study on reasons for declining or not completing MRI screening among women with an elevated breast cancer risk showed that the most important reason for not undergoing MRI was claustrophobia (11%) [16]. In our study, we did not use the word ‘claustrophobia’ but the description that ‘you have to lie in a small tunnel’, which was the most important disadvantage of MRI in both groups.

Outcomes of our study can be used in creating information brochures for women undergoing MRI screening, or even tailoring brochures and screening invitations to prior breast MRI screening experience of the women. The outcomes of our study and the outcomes of previous studies evaluating reasons for not participating can be used to inform women, especially those who have never had a breast MRI. Our findings suggest that women’s thoughts on MRI screening change after getting MRI screening. Future research is needed to evaluate the influence of preferences and perceptions on actual screening attendance outside a clinical study.

## 5 Conclusion

Our outcomes show that women in the FaMRIsc trial who received MRI screening, have a preference for this screening tool and that they have a lot of trust in the screening results of MRI. Women not undergoing MRI screening seem to be positive towards MRI screening as well but they also had considerable confidence in mammography screening. Overall, most women would accept a screening strategy with MRI as this was preferred the most. The way women think of MRI and mammography as screening tools depends on the screening strategy they are undergoing.

## Data availability statement

The data that support the findings of this study are available on request from the corresponding author (HAG). The data are not publicly available due to privacy or ethical restrictions.

## Funding

This work was funded by the Netherlands Organization for Health Research and Development (ZonMw grant no. 200320002). The funding source had no role in study design, data collection and analysis, decision to publish, or preparation of the manuscript.

## Ethical approval

Ethical approval for the FaMRIsc study has been granted by the Institutional Review Board of the Erasmus University Medical Centre, Rotterdam, the Netherlands.

## Declaration of competing interest

The authors declare no conflicts of interest.
