# Supplementary material for: Experiences, expectations and preferences regarding MRI and mammography as breast cancer screening tools in women at familial risk
Source: Breast. 2021 Jan 18;56:1–6. doi: 10.1016/j.breast.2021.01.002 (PMC7847961; doi:10.1016/j.breast.2021.01.002)
Supplement: Multimedia component 1 [file mmc1.docx]

**Experiences, expectations and preferences regarding MRI and mammography as breast cancer screening tools in women at familial risk**

# Supplementary appendix

Contents

[Co-authors of the FaMRIsc study group 2](#_Toc59543959)

[Questionnaire 4](#_Toc59543960)

[Outcomes of questions regarding breast cancer (screening) history of participants 8](#_Toc59543961)

[Outcomes of stratifications based on screening history 9](#_Toc59543962)

[Outcomes of questions not shown in the manuscript 10](#_Toc59543963)

## Co-authors of the FaMRIsc study group

**Writing committee**:

H Amarens Geuzinge (Erasmus University Medical Centre), Eveline AM Heijnsdijk (Erasmus University Medical Centre), A Inge-Marie Obdeijn (Erasmus University Medical Centre), Harry J de Koning (Erasmus University Medical Centre), Madeleine MA Tilanus-Linthorst (Erasmus University Medical Centre).

**Other members:**

Jan C Oosterwijk (Medical Centre Leeuwarden; University Medical Centre Groningen, the Netherlands), Ingeborg Mares-Engelberts, (Vlietland Ziekenhuis, Rotterdam, The Netherlands), Emiel JT Rutgers (The Netherlands Cancer Institute, Antoni van Leeuwenhoek Hospital, Amsterdam, the Netherlands); Sepideh Saadatmand (Erasmus University Medical Centre, Rotterdam, the Netherlands), Ritse M Mann (Radboud University Hospital, Nijmegen; The Netherlands Cancer Institute, Antoni van Leeuwenhoek Hospital, Amsterdam, the Netherlands), Rob AEM Tollenaar (Leiden University Medical Centre, Leiden, the Netherlands) Diderick BW de Roy van Zuidewijn (Medical Centre Leeuwarden, Leeuwarden, the Netherlands), Marc BI Lobbes (Maastricht University Medical Center, Maastricht, the Netherlands), Martijne van 't Riet (Reinier de Graaf Gasthuis, Delft, the Netherlands), Maartje J Hooning (Erasmus University Medical Centre, Rotterdam, the Netherlands), Margreet GEM Ausems (University Medical Centre Utrecht, Utrecht, the Netherlands), Claudette E Loo (The Netherlands Cancer Institute, Antoni van Leeuwenhoek Hospital, Amsterdam, the Netherlands), J Wesseling (The Netherlands Cancer Institute, Antoni van Leeuwenhoek Hospital, Amsterdam, the Netherlands), Ernest JT Luiten (University Medical Centre Utrecht, Utrecht, the Netherlands), Harmien M Zonderland (Amsterdam UMC, University of Amsterdam, the Netherlands), Cees Verhoef (Erasmus University Medical Centre, Rotterdam, the Netherlands), Carolien HM van Deurzen (Erasmus University Medical Centre, Rotterdam, The Netherlands), Eva Madsen (Erasmus University Medical Centre, Rotterdam The Netherlands), J Rothbarth (Erasmus University Medical Centre, Rotterdam, The Netherlands), Linetta B Koppert (Erasmus University Medical Centre Rotterdam, The Netherlands), Cecile de Monye (Erasmus University Medical Centre, Rotterdam, The Netherlands), Mandy M van Rosmalen (Erasmus University Medical Centre, Rotterdam, The Netherlands), Jolanda Remmelzwaal (The Netherlands Cancer Institute, Antoni van Leeuwenhoek Hospital, Amsterdam, The Netherlands), Margrethe Schlooz-Vries (Radboud University Hospital, Nijmegen, The Netherlands), Nico Karssemeijer (Radboud University Hospital, Nijmegen, The Netherlands), Roelie la Roi-Antonides (Medical Centre Leeuwarden, Leeuwarden, The Netherlands), Suzan van der Meij (Amsterdam University Medical Centre, Amsterdam, The Netherlands), Titia Lans (Amsterdam University Medical Centre, Amsterdam, The Netherlands), Wilma E Mesker (Leiden University Medical Centre, Leiden, The Netherlands), Kristien Keymeulen (Academic Hospital, Maastricht, The Netherlands), Wouter B Veldhuis (University Medical Centre Utrecht, The Netherlands), Arjen J Witkamp (University Medical Centre Utrecht, the Netherlands), Edith van Druten (Reinier de Graaf Gasthuis, Delft, The Netherlands), Eric Tetteroo (Amphia Ziekenhuis, Breda, The Netherlands), Carolien Contant (Maasstad ziekenhuis, Rotterdam, The Netherlands).

**Author contributions:**

HAG and MMAT-L developed the questionnaire and were responsible for the study design, HAG and EAMH did the data analyses, and made the first draft of the manuscript. HAG, EAMH, I-MO, HJdK, and MMAT-L were responsible for data interpretation and critical reading of the manuscript. All authors read and gave final approval of the submitted manuscript.

## Questionnaire

**
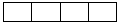
**

**In which year were you born?**

**Q1. In which screening group of the FaMRIsc-study did you participate?**

- The mammography group (annual mammography and clinical breast examination)
- The MRI group (annual MRI and clinical breast examination, biannual mammography)
- I did not participate in one of the above mentioned groups but I gave permission for my mammography screening results to be registered
- I did not participate in one of the above mentioned groups but I gave permission for my MRI screening results to be registered
- Other, …

**Q2. Have you been diagnosed with breast cancer?**

- No
- Yes, it was detected with screening as part of the FaMRIsc-study
- Yes, this was detected during the FaMRIsc-study but outside the study setting (for example is a different hospital, or at the national breast cancer screening programme)
- Yes, before the start of the FaMRIsc trial a pre-cancerous lesion has been detected
- I am currently receiving additional diagnostic testing due to a suspicious screening result of the FaMRIsc-study
- I am currently receiving additional diagnostic testing due to a suspicious finding which was detected outside the FaMRIsc-study
- Other, …

**Q3. Have you ever had a false alarm (you were referred for further diagnostic testing but it turned out not to be a cancer)?**

- No
- Yes

**Q4. If you were not participating in the MRI-group of the FaMRIsc-study, have you have had a breast MRI in your life?**

- No
- Yes

**Q5. I expect that by screening the chance that breast cancer will be detected early and therefore be curable:**

- Is much higher than without screening
- Is a little higher than without screening
- Is not higher than without screening

**Q6. I prefer screening:**

- With mammography
- With MRI
- With both mammography and MRI
- In the national breast cancer screening program
- No preference
- No screening at all
- Other, …

**Q7. After only clinical breast examination, do you trust that the findings (that you do or do not have breast cancer) are correct? Please circle the number that best represents your opinion:**

0------------1------------2------------3------------4
No trust A lot of trust

**Q8. After only mammography, do you trust that the findings (that you do or do not have breast cancer) are correct? Please circle the number that best represents your opinion:**

0------------1------------2------------3------------4
No trust A lot of trust

**Q9. After only MRI, do you trust that the findings (that you do or do not have breast cancer) are correct? Please circle the number that best represents your opinion:**

0------------1------------2------------3------------4
No trust A lot of trust

**Q10. I expect that the chance of detecting breast cancer early by MRI […] is than with mammography**

- Much smaller
- Smaller
- Similar
- Slightly higher
- Much higher

**Q11. Advantages of mammography are for me […]
(Please provide at ‘Ranking’ with number 1 which advantage is of most importance for you).**

**Ranking**

- High chance of early detection of breast cancer …
- It does not take much time …
- You can get the screening result quickly …
- It has a small chance of a false alarm …
- I can get it close to where I live …
- It is not expensive …
- I was already familiar with mammography …
- I do not see advantages of mammography …
- Other, … …

**Q12. Disadvantages of mammography are for me […]
(Please provide at ‘Ranking’ with number 1 which advantage is of most importance for you).**

**Ranking**

- It is painful …
- You get X-radiation …
- It does not detect all breast cancers …
- It sometimes causes a false alarm …
- It takes (too) much time …
- It takes long before I get the result …
- I have to take off my clothes …
- I do not see disadvantages of mammography …
- Other, … …

**Q13. Advantages of MRI are for me […]
(Please provide at ‘Ranking’ with number 1 which advantage is of most importance for you).**

**Ranking**

- High chance of early detection of breast cancer …
- You don’t get X-radiation …
- You can get the screening result quickly …
- It has a small chance of a false alarm …
- It does not cause pain …
- I can keep some clothes on …
- Other, … …

**Q14. Disadvantages of MRI are for me […]
(Please provide at ‘Ranking’ with number 1 which advantage is of most importance for you).**

**Ranking**

- The infusion of contrast fluid is unpleasant …
- You have to lie in a small tunnel …
- The noise is unpleasant …
- It takes a lot of time …
- It does not detect all breast cancers …
- It sometimes causes a false alarm …
- Some contrast fluid may remain in my body, even

though no side effects of this are known …

- It is far from home which causes travel time …
- I have to wait more than one day for the result ...
- It is expensive …
- I do not see disadvantages of MRI …
- Other, … …

**Q15. I would only have a preference for MRI if it, in comparison with mammography, at least … (please choose one answer):**

- Detects breast cancer at an early stage as often as mammography
- Detects breast cancer twice as often as mammography at an early stage
- Detects breast cancer three times as often as mammography at an early stage
- I never prefer MRI
- I always prefer MRI

**Q16. I prefer MRI screening, even if, in comparison with mammography… (please choose one answer):**

- It causes as often a false alarm as mammography
- It causes a false alarm twice as often as mammography
- It causes a false alarm three times as often as mammography
- I never prefer MRI
- I always prefer MRI

**Q17. I prefer MRI screening, even if, in comparison with mammography… (please choose one answer):**

- It has the same price
- Also if MRI is twice as expensive
- Also if MRI is five times as expensive
- The price is not important to me
- I never prefer MRI
- I always prefer MRI

**Here is room for comments about breast cancer screening that were not captures in above questions, but that you find important:**

## Outcomes of questions regarding breast cancer (screening) history of participants

**Table S1.** Answers to question 1: In which screening group of the FaMRIsc-study did you participate?

|  | Total group (N=255) |
| --- | --- |
| The mammography group (annual mammography and clinical breast examination) | 136 (53.3%) |
| The MRI group (annual MRI and clinical breast examination, biannual mammography) | 105 (41.2%) |
| I did not participate in one of the above mentioned groups but I gave permission for my mammography screening results to be registered | 9 (3.5%) |
| I did not participate in one of the above mentioned groups but I gave permission for my MRI screening results to be registered | 3 (1.2%) |
| Other, … | 2 (0.8%) |

**Table S2.** Answers to question 2: Have you been diagnosed with breast cancer?

|  | MRI-group (N=108) | Mammography-group (N=145) |
| --- | --- | --- |
| No | 97 (89.8%) | 137 (94.5%) |
| Yes, it was detected with screening as part of the FaMRIsc-study | 4 (3.7) | 2 (1.4%) |
| Yes, this was detected during the FaMRIsc-study but outside the study setting (for example is a different hospital, or at the national breast cancer screening programme) | 1 (0.9%) | 0 |
| Yes, before the start of the FaMRIsc trial a pre-cancerous lesion has been detected | 1 (0.95) | 3 (2.1%) |
| I am currently undergoing additional diagnostic testing due to a suspicious screening result of the FaMRIsc-study | 0 | 0 |
| I am currently undergoing additional diagnostic testing due to a suspicious finding which was detected outside the FaMRIsc-study | 2 (1.9%) | 0 |
| Other, … | 3 (2.8%) | 3 (2.1%) |

**Table S3.** Answers to question 3: Have you ever had a false alarm?

|  | MRI-group (N=108) | Mammography-group (N=145) |
| --- | --- | --- |
| No | 68 (63.0%) | 104 (71.7%) |
| Yes | 40 (37.0%) | 41 (28.3%) |

**Table S4.** Answers to question 4: If you were not participating in the MRI-group of the FaMRIsc-study, have you have had a breast MRI in your life?

|  | Mammography-group (N=145) |
| --- | --- |
| No | 89 (61.4%) |
| Yes | 49 (33.8%) |
| Missing | 7 (4.8%) |

## Outcomes of stratifications based on screening history

**Table S5.** Answers to the question ‘Do you trust in the findings that you do/do not have breast cancer after only MRI’, by prior experience with MRI

|  | Mammography-group (N=145)* | |
| --- | --- | --- |
|  | **Prior experience MRI (n=49)** | **No prior experience MRI (n=89)** |
| No trust | 0 | 0 |
| A little trust | 2 (4%) | 0 |
| Neutral | 4 (8%) | 26 (29%) |
| Quite some trust | 4 (8%) | 8 (9%) |
| A lot of trust | 38 (78%) | 45 (51%) |
| Missing | 1 (2%) | 10 (11%) |

*7 women did not answer the question about prior experience with MRI

**Table S6.** Preference for screening modality, by screening group and by experience of a false alarm

|  | MRI-group (N=108) | | Mammography-group (N=145) | |
| --- | --- | --- | --- | --- |
| Preference for screening modality | **False alarm ever (n=40)** | **False alarm never (n=68)** | **False alarm ever (n=41)** | **False alarm never (n=104)** |
| Mammography | 0 | 6 (9%) | 11 (27%) | 34 (33%) |
| MRI | 18 (45%) | 23 (34%) | 9 (22%) | 17 (16%) |
| Mammography and MRI | 19 (48%) | 35 (52%) | 15 (37%) | 35 (34%) |
| In the national breast cancer screening program | 0 | 0 | 1 (2%) | 4 (4%) |
| No preference | 3 (8%) | 3 (4%) | 1 (2%) | 7 (7%) |
| No screening at all | 0 | 0 | 0 | 0 |
| Other, … | 0 | 1 (2%) | 3 (7%) | 6 (6%) |
| Missing | 0 | 0 | 1(2%) | 1 (1%) |

*p*-value MRI-group: 0.29; *p*-value Mammography-group: 0.72

**Table S7.** Preference of screening modality, by prior experience with MRI

|  | Mammography-group (N=145)* | |
| --- | --- | --- |
| Preference for screening modality | **Prior experience MRI (n=49)** | **No prior experience MRI (n=89)** |
| Mammography | 14 (29%) | 29 (33%) |
| MRI | 9 (18%) | 16 (18%) |
| Mammography and MRI | 19 (39%) | 27 (30%) |
| In the national breast cancer screening program | 3 (6%) | 2 (2%) |
| No preference | 1 (2%) | 7 (8%) |
| No screening at all | 0 | 0 |
| Other, … | 2 (4%) | 7 (8%) |
| Missing | 1 (1%) | 1 (1%) |

*7 women did not answer the question about prior experience with MRI

*p*-value: 0.53

## Outcomes of questions not shown in the manuscript

**Table S8.** Answers to question 5: I expect that the chance of detecting breast cancer in an early stage and therefore better curable, is […] with screening than without screening.

|  | MRI-group (N=108) | Mammography-group (N=145) |
| --- | --- | --- |
| Much higher | 88 (81.5%) | 116 (80.0%) |
| A little higher | 18 (16.7%) | 27 (18.6%) |
| Not higher | 2 (1.9%) | 2 (1.4%) |

**Table S9.** Answers to question 7: After only clinical breast examination, do you trust that the findings (that you do or do not have breast cancer) are correct?

|  | MRI-group (N=108) | Mammography-group (N=145) |
| --- | --- | --- |
| No trust | 20 (18.5%) | 16 (11.0%) |
| A little trust | 41 (38.0%) | 40 (27.6%) |
| Neutral | 14 (13.0%) | 14 (9.7%) |
| Quite some trust | 26 (24.1%) | 53 (36.6%) |
| A lot of trust | 7 (6.5%) | 21 (14.5%) |
| Missing | 0 | 1 (0.7%) |

**Table S10.** Answers to question 15: I would only have a preference for MRI if it, in comparison with mammography at least […]

|  | MRI-group (N=108) | Mammography-group (N=145) |
| --- | --- | --- |
| Detects breast cancer as often in an early stage as mammography | 35 (32.4%) | 38 (26.2%) |
| Detects breast cancer twice as often in an early stage as mammography | 26 (24.1%) | 54 (37.2%) |
| Detects breast cancer three times as often in an early stage as mammography | 7 (6.5%) | 17 (11.7%) |
| I never prefer MRI | 0 | 9 (6.2%) |
| I always prefer MRI | 39 (36.1%) | 23 (15.9%) |
| Missing | 1 (0.9%) | 4 (2.8%) |

**Table S11.** Answers to question 16: I prefer MRI screening, even if, in comparison with mammography…

|  | MRI-group (N=108) | Mammography-group (N=145) |
| --- | --- | --- |
| It causes as often a false alarm as mammography | 39 (36.1%) | 60 (41.4%) |
| It causes twice as often a false alarm as mammography | 8 (7.4%) | 12 (8.3%) |
| It causes three times as often a false alarm as mammography | 8 (7.4%) | 4 (2.8%) |
| I never prefer MRI | 8 (7.4%) | 28 (19.3%) |
| I always prefer MRI | 42 (38.9%) | 29 (20.0%) |
| Missing | 3 (2.8%) | 12 (8.3%) |

**Table S12.** Answers to question 17: I prefer MRI screening, even if, in comparison with mammography…

|  | MRI-group (N=108) | Mammography-group (N=145) |
| --- | --- | --- |
| It has the same price | 16 (14.8%) | 23 (15.9%) |
| Also if MRI is two times as expensive | 7 (6.5%) | 5 (3.4%) |
| Also if MRI is five times as expensive | 2 (1.9%) | 1 (0.7%) |
| The price is not important to me | 43 (39.8%) | 60 (41.4%) |
| I never prefer MRI | 5 (4.6%) | 27 (18.6%) |
| I always prefer MRI | 32 (29.6%) | 20 (13.8%) |
| Missing | 3 (2.8%) | 9 (6.2%) |
